# Prevalence of Abuse in Elders With Psychiatric Morbidity and Its Sociodemographic Association

**DOI:** 10.7759/cureus.7906

**Published:** 2020-04-30

**Authors:** Aarushi Sudan, Pratyush Shahi, Dhawani Julka

**Affiliations:** 1 Psychiatry, University College of Medical Science and Guru Teg Bahadur Hospital, Delhi, IND; 2 Orthopaedics, University College of Medical Sciences and Guru Teg Bahadur Hospital, Delhi, IND; 3 Medicine, University College of Medical Sciences, New Delhi, IND

**Keywords:** elder abuse, neglect, psychiatric illness, risk factors, elderly

## Abstract

Objective

Our aim in this study is to assess the prevalence of abuse in elders with psychiatric illness and its association with various sociodemographic variables.

Methods

This cross-sectional comparative study included 300 elderly (aged more than 65 years) patients divided into two groups. Group 1 consisted of 150 patients with psychiatric illnesses presenting to the psychiatry outpatient department (OPD), whereas group 2 comprised 150 patients with somatic illnesses presenting to the OPDs of other departments. Elder Abuse Suspicion Index (EASI) was used as a screening tool for the detection of elder abuse. In patients with suspicion of abuse on EASI, the Actual Abuse Tool was used for confirmation and assessment.

Results

A significantly higher prevalence of abuse was seen in elders with psychiatric illness (21.3%) compared to those with somatic illness (4%). Among sociodemographic variables, a significant correlation was found between elder abuse and gender, literacy, and marital status.

Conclusions

Elder abuse is a serious social problem. Awareness should be raised to improve the attitude and behavior towards seniors. Healthcare professionals, especially in the psychiatry field, should be made more capable of and open towards early detection of and intervention against elder abuse. Further research on this topic in India is highly recommended.

## Introduction

The elderly population (people aged more than 65 years) is growing both in number and proportion worldwide as a result of increased life expectancy. This accelerated growth has led to a rise in concerns regarding lack of attention towards this group, its members being more prone to chronic illnesses, dependence, and abuse.

A relatively new negative attitude towards seniors is a worrying trend. It ranges from ageism to elder abuse. The World Health Organisation (WHO) defines elder abuse as a single or repeated act or lack of appropriate action, occurring within any relationship where there is an expectation of trust, which causes harm or distress to an older person [1]. Physical or mental disability and challenging behavior of the elderly, history of domestic violence in the family, and pathological behavior of the carer are the causative factors of elder mistreatment [2]. Elder abuse can be psychological, physical, financial, or sexual, or one characterized by neglect; and it can be intentional or unintentional [3]. It leads to grave consequences such as physical and emotional suffering, hospital visits and institutionalization, and even mortality [4].

Studies have been done to assess if psychiatric illness in the geriatric population leads to an increased vulnerability to abuse; however, none have been conducted in the Indian setting so far. Through our study, we aimed to rectify this shortcoming. We also analyzed the association of elder abuse with various sociodemographic variables.

## Materials and methods

Study design

This cross-sectional comparative study was conducted at a tertiary care hospital. It included 300 elderly patients divided into two groups. Group 1 consisted of 150 patients with psychiatric illnesses, whereas group 2 was comprised of 150 patients with somatic ailments. Elder Abuse Suspicion Index (EASI) was used as a screening tool for the detection of elder abuse [5]. In patients with suspicion of abuse on EASI, the Actual Abuse Tool was used for confirmation and assessment [6].

Inclusion criteria

Patients of either sex who were above the age of 65 years presenting to the psychiatry outpatient department (OPD) and OPDs of other departments with psychiatric and somatic illnesses, respectively, were included in the study.

Exclusion criteria

Patients with a medical or surgical emergency, terminal end-stage disease, severe neurocognitive disorder, and those unwilling to participate in the study were excluded.

Ethical issues

All the subjects were explained about the aims and objectives of the study and were included in the study only after receiving informed consent. Anonymity and confidentiality were maintained. The dignity of the participants was respected.

Statistical analysis

For statistical analysis, the chi-squared test and SPSS Statistics version 15.0 (IBM, Armonk, NY) were used. A p-value of <0.05 was considered statistically significant.

## Results

The prevalence of abuse was found to be 21.3% in group 1 and 4% in group 2 (Table 1). The difference was statistically significant (p<0.05). In total, there were 38 cases of reported abuse. In both groups, the most common type of abuse was psychological (Table 2).

**Table 1:** Prevalence of abuse in elders in group 1 and group 2 *Patients with psychiatric illnesses **Patients with somatic illnesses

Group	Abuse reported, n	Abuse not reported, n	Prevalence of abuse, %
Group 1* (n = 150)	32	118	21.3%
Group 2** (n = 150)	6	144	4%

**Table 2:** Types of abuse in elders in group 1 and group 2 *Patients with psychiatric illnesses **Patients with somatic illnesses

Type of abuse	Group 1* (n = 32), n (%)	Group 2** (n = 6), n (%)
Psychological	15 (46.8%)	3 (50%)
Neglect	6 (18.7%)	2 (33.3%)
Exploitation	4 (12.5%)	0
Physical	1 (3.1%)	0
Mixed	6 (18.7%)	1 (16.7%)

In the 38 reported cases of abuse, an assessment based on sociodemographic variables (age, gender, religion, literacy, residence, marital status, family structure, and socioeconomic status) was performed (Table 3). Among these, on applying the chi-squared test, a statistically significant (p<0.05) correlation was found between abuse and gender, literacy, and marital status.

**Table 3:** Association of elder abuse with sociodemographic variables

Sociodemographic variables	Abuse reported (n = 38), n (%)
Age, years	
60–69	19 (50%)
70–79	9 (23.7%)
>80	10 (26.3%)
Gender	
Male	6 (15.8%)
Female	32 (84.2%)
Literacy	
Illiterate	28 (73.6%)
Literate	10 (26.4%)
Residence	
Rural	17 (44.7%)
Urban	21 (55.3%)
Marital status	
Married	12 (31.7%)
Widow/widower	26 (68.3%)
Family structure	
Joint	22 (58%)
Nuclear	16 (42%)
Socioeconomic status	
High	10 (26.3%)
Middle	12 (31.7%)
Low	16 (42%)

## Discussion

In traditional Indian society, elders held an important place and were looked at with a sense of respect. Lately, due to Westernization, migration from rural to urban areas, and increasing work-related stress, the younger generation is increasingly viewing the elders more as a burden and is developing a negative approach towards them [7]. This has led to the rise of elder abuse, a concept previously alien to the Indian society. Although there is an abundance of data on population ageism in India, elder abuse has been rarely researched upon [8]. Having a physical or mental comorbidity has been reported to increase the susceptibility of elders to abuse. A few studies from the West have highlighted a higher prevalence of abuse in elders with psychiatric illnesses [9]. Through this study, we aimed to study this correlation in the Indian setting and highlight the sociodemographic contributory factors to elder abuse as well.

In this study, a significantly higher prevalence of abuse was seen in elders with psychiatric illnesses (21.3%) when compared to those with somatic illnesses (4%). Luzny et al. had a similar finding in their study in the Czech Republic [9]. This can be attributed to the stigma attached to psychiatric illnesses. Neuropsychiatric symptoms like psychosis, overactivity, aggression, depression, and anxiety in such patients can lead to challenging behavior, causing frustration among the caregivers, often leading to a point where they no longer care [10]. This can lead to a vicious cycle where abuse imparts a feeling of neglect, loneliness, and fear in the elderly and can cause aggravation of their psychiatric illness.

Psychological abuse was the most common form of abuse followed by the neglect of elders with psychiatric illnesses (46.8%) as well as those with somatic illnesses (50%). Most of the studies conducted previously had reported similar findings [11-14]. In our study, only one patient reported physical abuse, and none reported sexual abuse. Under-reporting of sexual abuse in this study, as well as in previous studies, can be due to a feeling of embarrassment and fear of further abuse [15].

Among sociodemographic variables, a significant correlation was found between elder abuse and gender, literacy, and marital status. However, no correlation was found with age, religion, residence, family structure, and socioeconomic status. Patel et al. have found a positive correlation between abuse and literacy, marital status, family type, and severity of depression in their study on elders with depression [15]. The majority of the subjects reporting abuse were females (84.2%). Older females in present-day India come from a largely patriarchal society where they were seen merely as caregivers and had no financial independence. This might explain the higher prevalence of abuse among women.

Illiteracy was found to be significantly associated with elder abuse in this study. Other studies from India had noted a similar correlation [16,17]. The reason for this might be carers’ perception of less worth and value towards these patients and a resultant negative approach towards them. Widows and widowers were found to suffer from significantly more abuse than married elders (68.3% vs. 31.7%) in our study. This is consistent with the findings of Patel et al. [15]. According to the WHO, elderly females are at a higher risk of financial abuse like the seizure of the property after they are widowed.

Our study has some limitations. Being a cross-sectional study, it lacked any intervention and follow-up. Also, we did not analyze whether the abuse was aggravating the pre-existing psychiatric illnesses among the participants. We recommend future interventional studies where the patients can be followed up to assess the efficacy of the intervention.

Elder abuse law in India

The Maintenance and Welfare of Parents and Senior Citizens Act was passed by the Government of India in 2007 [18]. It makes providing maintenance to the parents a legal obligation to children and thereby aims to prevent elder abuse. Under the Act, the financially-dependent elders can demand maintenance from their children and grandchildren. Childless senior citizens can demand maintenance from their relatives. The Act was amended in 2019 to include stepchildren, adoptive children, and children-in-law under the definition of “children” and parents-in-law and grandparents under the definition of “parents” [19]. However, the awareness regarding the existence of this Act is very low, with only 14% of elder abuse victims knowing about it [20].

Recommendations to curb elder abuse

**Figure 1:**
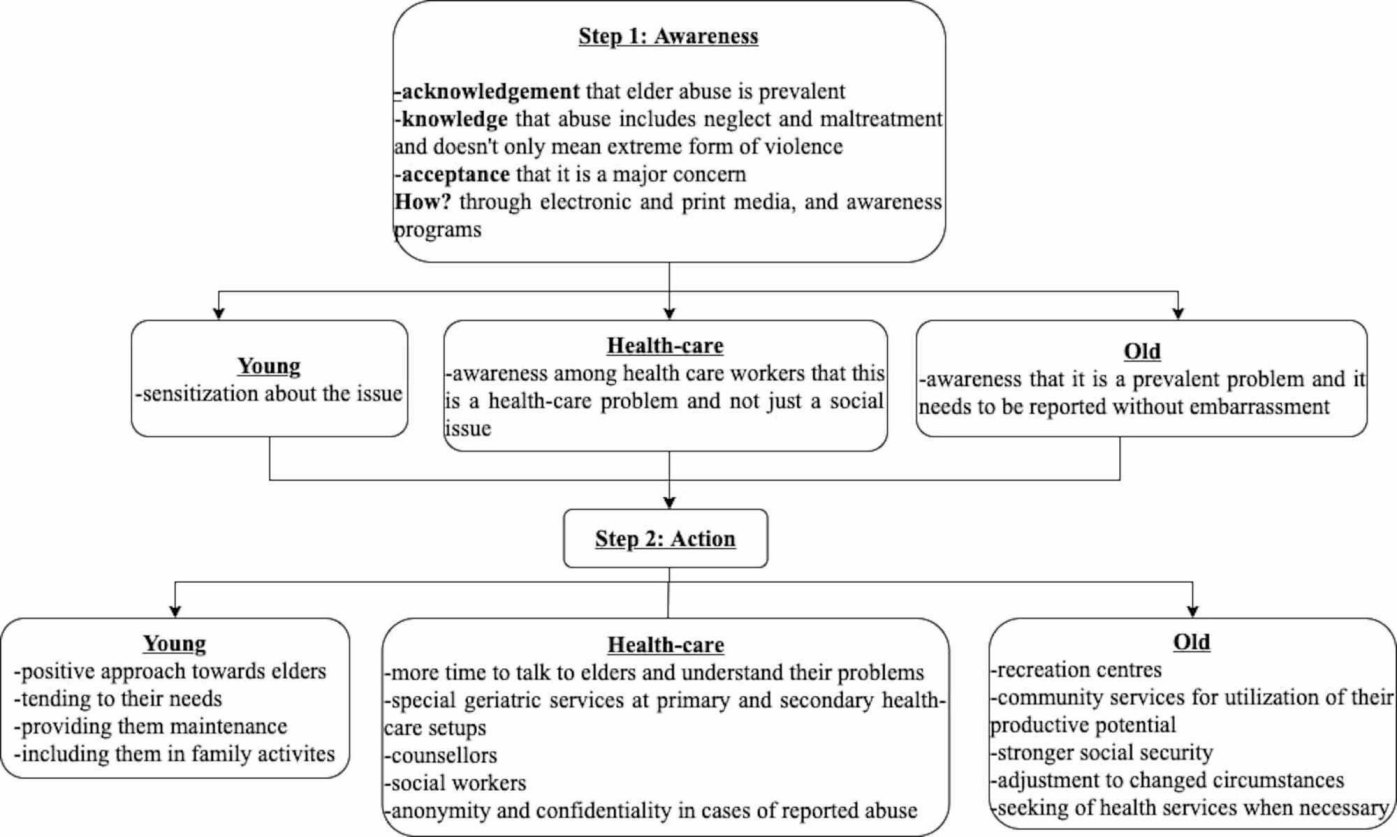
Two-step algorithm to curb elder abuse

We recommend a two-step approach to curb elder abuse, which is represented in the algorithm below. It revolves around two factors: awareness and action (Figure 1). We believe that the implementation of the steps delineated in the algorithm will contribute immensely towards the efforts to curb elder abuse.

## Conclusions

Elder abuse is a serious social issue with devastating consequences such as physical and emotional suffering, hospital visits and institutionalization, and even mortality. Our study found that the prevalence of elder abuse is significantly associated with psychological morbidity and certain sociodemographic factors. Raising awareness about this problem and taking proper actions are necessary to curb this malaise. Further research on this topic in India through interventional studies with follow-ups is strongly recommended.
